# Brazilian cohorts with potential for life-course studies: a scoping review

**DOI:** 10.11606/s1518-8787.2020054001825

**Published:** 2020-05-15

**Authors:** Waleska Regina Machado Araujo, Iná S. Santos, Naercio Aquino Menezes, Maria Thereza Costa Coelho de Souza, Antonio Jose Ledo Alves da Cunha, Alicia Matijasevich

**Affiliations:** I Universidade de São Paulo. Faculdade de Medicina Programa de Pós-Graduação em Saúde Coletiva São PauloSP Brasil Universidade de São Paulo. Faculdade de Medicina. Programa de Pós-Graduação em Saúde Coletiva. São Paulo, SP, Brasil; II Pontifícia Universidade Católica do Rio Grande do Sul Programa de Pós-Graduação em Pediatria e Saúde da Criança Porto AlegreRS Brasil Pontifícia Universidade Católica do Rio Grande do Sul. Programa de Pós-Graduação em Pediatria e Saúde da Criança. Porto Alegre, RS, Brasil; III Universidade de São Paulo Faculdade de Economia e Administração Departamento de Economia. São PauloSP Brasil Universidade de São Paulo. Faculdade de Economia e Administração. Departamento de Economia. São Paulo, SP, Brasil; IV Universidade de São Paulo Instituto de Psicologia Departamento de Psicologia da aprendizagem, do desenvolvimento e da personalidade São PauloSP Brasil Universidade de São Paulo. Instituto de Psicologia. Departamento de Psicologia da aprendizagem, do desenvolvimento e da personalidade. São Paulo, SP, Brasil; V Universidade Federal do Rio de Janeiro Faculdade de Medicina Departamento de Pediatria Rio de JaneiroRJ Brasil Universidade Federal do Rio de Janeiro. Faculdade de Medicina. Departamento de Pediatria. Rio de Janeiro, RJ, Brasil; VI Universidade de São Paulo Faculdade de Medicina Departamento de Medicina Preventiva São PauloSP Brasil Universidade de São Paulo. Faculdade de Medicina. Departamento de Medicina Preventiva. São Paulo, SP, Brasil

**Keywords:** Cohort studies, Child development, Health development, Analytical Epidemiology, Bibliometrics, Systematic Review

## Abstract

**OBJECTIVE:**

To identify the Brazilian cohorts that started either in the prenatal period or at birth, to describe their characteristics and the explored variables, and to map the cohorts with potential for studies on early determinants on health and the risk of falling ill on later stages of the life cycle.

**METHODS:**

A scoping review was carried out. The articles were searched in the electronic databases PubMed and Virtual Health Library (VHL). The descriptors used were [(((“Child” OR “Child, Preschool” OR “Infant” OR “Infant, Newborn”) AND (Cohort Studies” OR “Longitudinal Studies”)) AND “Brazil”)]. The inclusion criteria were Brazilian cohorts that started the baseline in the prenatal period or at birth and with at least two follow-ups with the participants. In order to meet the concept of LCE, we excluded those cohorts whose follow-ups were restricted to the first year of life, as well as those that did not address biological, behavioral and psychosocial aspects, and cohorts with data collection of a single stage of the life cycle.

**RESULTS:**

The search step identified 5,010 articles. Eighteen cohorts were selected for descriptive synthesis. The median number of baseline participants was 2,000 individuals and the median age at the last follow-up was 9 years. Sample loss at the last follow-up ranged from 9.2 to 87.5%. Most cohorts monitored two phases of the life cycle (the perinatal period and childhood). The Southern region had the highest number of cohorts. The main variables collected were sociodemographic and environmental aspects of the family, morbidity aspects, nutritional practices and lifestyle.

**CONCLUSIONS:**

We recommend the continuity of these cohorts, the approach to different social contexts and the performance of follow-ups with participants in different phases of the life cycle for the strengthening and expansion of life course epidemiology analyses in Brazil.

## INTRODUCTION

Life course epidemiology (LCE) is a field that compromises the studies of biological, behavioral and psychosocial processes that occur throughout the life of individuals. It links the health conditions and the risk of illness of an adult to physical or psychosocial exposures that might have happened during pregnancy, infancy or adolescence, in the earlier stages of life or through generations^1^. Its purpose is to build and test theoretical models that might postulate paths that link exposures at different stages of the life course to later health outcomes^2^. These models explore the time and interactions of biological and psychosocial exposures to identify risks and protection processes throughout the life course^3,4^.

As such, cohort studies are a valuable tool for LCE, as they follow their participants from exposure to the occurrence of outcomes of interest^5^. These studies are generally conducted to assess the natural history of diseases, estimate disease frequency measures (incidence), identification of risk factors for health problems, prognosis and survival analysis^6,7^. Cohorts that collect information at different stages of their participants’ lives and with medium and long-term outcomes are excellent for testing explanatory models of LCE^8,9^, and it is possible to explore risk accumulation models and critical periods, in addition to investigating the mediating and modifying factors of the association between exposures and outcomes in study^2,3^.

Cohorts that start in the prenatal period or at birth offer a unique opportunity to assess the influence of early exposures on health throughout life^4^. They are mainly concentrated in high-income countries and differ from cohorts conducted in middle and low income countries by the greater number of follow-ups, follow-up of participants for longer periods and greater sophistication of the measures performed^10^. These cohorts, in middle- and low-income countries, offer valuable contributions due to the diversity of exposures and the different structures for disease determination^11^. In countries such as Brazil, with cultural diversity and regional inequality, they can offer contributions as valuable as cohorts from high-income countries. However, the research gaps of these studies in the country are not mapped, and a synthesis of information with the characteristics and outcomes studied by the main cohorts of this type is important in order to advance the studies of life course epidemiology throughout the country.

This study aims to describe the cohort studies initiated in the prenatal period or at birth conducted in Brazil, with the potential to study the early determinants of health and disease and the risk of falling ill in later stages of the life cycle. Through a scoping review, it was possible to describe the main methodological characteristics of these cohorts, as well as the variables studied, the location in the country and the needs related to epidemiological research of the life cycle in Brazil.

## METHOD

We conducted a scoping review to identify and map the available evidence on Brazilian cohorts with potential for life course epidemiology studies. This review is conducted to summarize and disseminate the research results and recognize research gaps in the available literature^12^. We followed the following steps: identification of the research question (“What are the characteristics of the Brazilian cohorts started at birth and in the prenatal period with the potential to study the early determinants of health and disease and the risk of falling ill in later stages of the life cycle?”), identification of relevant articles, selection of studies, data mapping and compilation, summary and reporting of results. The articles were searched in the electronic databases PubMed and Virtual Health Library (VHL). The descriptors were selected at the Medical Subject Headings (MeSH) with the following combination: [(((Child” OR “Child, Preschool” OR “Infant” OR “Infant, Newborn”) AND (Cohort Studies” OR “Longitudinal Studies”)) AND “Brazil”)]. The search strategy was adapted for each database. All articles published as of June 16, 2018 were considered in the review.

The review included cohort studies conducted in Brazil that started the baseline of the study in the prenatal period or at birth and with at least two follow-ups with the participants. The studies excluded were those that described only the baseline and not the follow-ups performed. In order to meet the concept of LCE, we excluded those cohorts whose follow-ups were restricted to the first year of life, as well as those that did not address biological, behavioral and psychosocial aspects, and cohorts with data collection of a single stage of the life cycle. We considered as stages of the life cycle the perinatal period (between the 22nd week of gestation and six days of life), childhood (between the first week of life and six years of age), school age (between 6 and 12 years), adolescence (between 12 and 18 years) and adulthood (18 years or more)^13^. Two reviewers selected the titles and abstracts of the articles and read the full text of the eligible articles. Given that the scoping review aimed to map the evidence on a relevant topic, an evaluation of the quality of the evidence included in the review was not necessary^12^. Finally, from the interpretation of the results of this review, we presented recommendations to advance the studies of life course epidemiology in Brazil.

The following information was extracted from the selected studies: geographic location of the cohort (city, state, region); year of beginning of the cohort (baseline); type of cohort according to the age of baseline participants, divided into birth (recruitment of participants started at birth) and prenatal (recruitment initiated during pregnancy); type of cohort regarding the time of occurrence of exposure and outcome and the beginning of the study, divided into prospective (exposure data were collected at the beginning of the study and the outcome has not yet occurred), retrospective (exposure and outcome were defined based on the data that occurred before the beginning of the study, both in the past) and ambispective (exposure data were collected in the past and the outcome has not yet occurred); type of data used in the cohort, divided into primary (data collected for the first time, to meet the needs of the current cohort) and secondary (information obtained from previously collected data, which was not collected at the time of the research); reference base of the sample, divided into hospital-based (individuals of a hospital or health center) and population-based (representative or convenience); number of participants in the baseline; number of participants in the last follow-up; average age of the participants in the last follow-up; total number of follow-ups with participants; mean age of participants in each follow-up; information collected at each stage of the life cycle.

These data were complemented with information extracted from publications from the list of references of selected articles, as well as from electronic pages of cohort studies, from contacting via e-mail researchers of these studies and consultations with databases of dissertations and theses. Finally, a descriptive analysis of the main characteristics of the cohorts included in the review was performed.

## RESULTS

### Search Results and Article Selection

**Figure 1:**
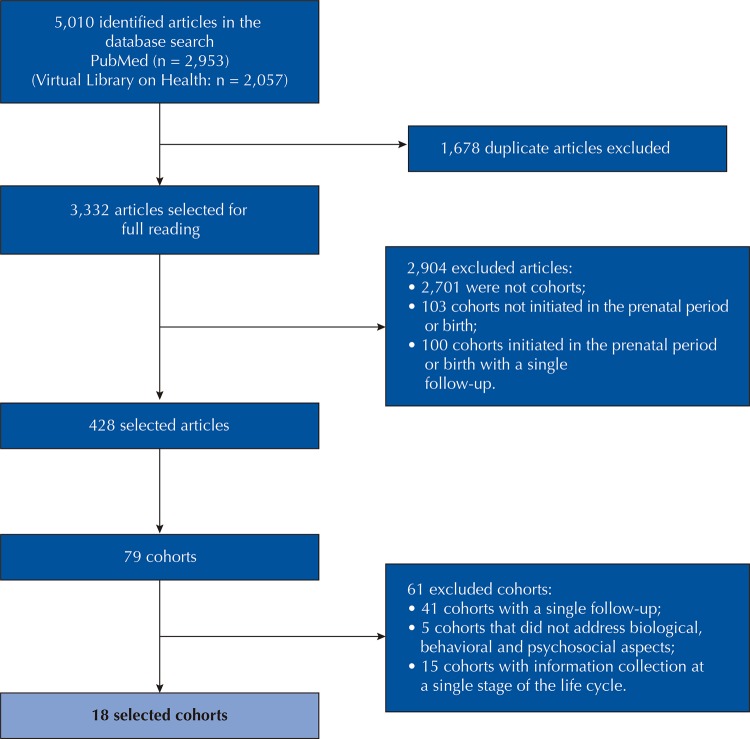
Flowchart of the search stage of the article selection in the systematic review.

The search stage identified 5,010 articles in the databases, excluding 1,678 duplicated ones. After reading the titles and abstracts, 2,904 articles were excluded because they did not meet the eligibility criteria. After reading the full text, 428 articles from 79 cohort studies conducted in Brazil initiated in the prenatal period or at birth with at least two follow-ups with the participants were identified. Of these 79 cohorts, 41 with follow-ups only during the first year of life and five that did not address biological, behavioral and psychosocial aspects were excluded. Due to the interest in analyzing cohorts that explored effects of exposures that occurred in the perinatal period, childhood, adolescence and in later stages of the life cycle, fifteen cohorts with data collection of a single stage of the life cycle were excluded. At the end, 18 cohorts were selected (n = 345 articles). The flowchart of the review steps is illustrated in Figure 1.

### Description of Selected Cohorts

Of the 18 cohorts identified^14^, 15 started at birth^14^ and three, at the prenatal period^23,30,31^. All cohorts were prospective and collected primary data. Ten cohorts^15,18,20^ had a representative sample of the baseline reference population, seven cohorts^14,16,17,19,27,28,30^ used a convenience sample, and a single cohort was hospital-based^23^. The number of baseline participants ranged from 189 to 7,747 individuals (median of 2,000). The mean age at the last follow-up ranged from 2 to 30 years (median of 9 years) and the number of follow-ups ranged from 2 to 11 (median of 4), as observed in Table 1. Sample loss in the last follow-up of the cohorts ranged from 9.2 to 87.5% (median of 42.0%), and the main difficulties reported by the authors were the not knowing the whereabouts of the participants, loss of telephone contact and change of address.

**Table 1:** Characteristics of the 18 cohorts selected, by geographic region of Brazil.

Cohort location	Baseline cohort year	Age of participants at baseline	Sample reference base	Number of baseline participants		Number of participants in the last follow-up	Mean age at last follow-up (years)	Total number of follow-ups	Loss to follow-up^1^
Northern region									
Itaituba – PA^14^	2000–2001	Birth	Population sample	239		90	8	3	59.3
Cruzeiro do Sul – AC^15^	2015–2016	Birth	Representative urban population	1,195		853	2	3	28.6
Northeast region									
Fortaleza – CE^16^	1989–1993	Birth	Population sample	189	77		9.6	9	59.3
Cities of Pernambuco^17^	1993–1994	Birth	Population sample	549	217		18	8	60.5
São Luís – MA^18^	1997–1998	Birth	Representative population	2,691	664		18	2	75.3
Feira de Santana – BA^19^	2004–2005	Birth	Population sample	1,309	672		6	10	48.7
São Luís – MA^20^	2010	Birth	Representative population	5,166	3,306		2	2	36
Southeast region									
Ribeirão Preto – SP^21^	1978–1979	Birth	Representative population	6,764	2,063		24	3	69.5
Ribeirão Preto – SP^22^	1994	Birth	Representative population	2,846	622		23	2	78.1
Ribeirão Preto – SP^23^	2001	Prenatal care	Hospital	449	56		12	2	87.5
Ribeirão Preto – SP^24^	2010	Birth	Representative population	7,747	4,182		2	2	46
South region									
Pelotas – RS^25^	1982	Birth	Representative population	5,914	3,701		30	10	37.4
Pelotas – RS^26^	1993	Birth	Representative population	5,249	4,106		18	11	21.8
São Leopoldo – RS^27^	2001–2002	Birth	Population sample	500	307		7.5	4	38.6
Pelotas – RS^28^	2002–2003	Birth	Population sample	973	616		8	4	36.7
Pelotas – RS^29^	2004	Birth	Representative population	4,231	3,563		11	7	15.8
Porto Alegre – RS^30^	2008	Prenatal care	Population sample	715	475		3.2	3	33.6
Pelotas – RS^31^	2015	Prenatal care	Representative population	4,426	4,018		2	4	9.2

^1^ Percentage of participants who were not followed in the last follow-up in relation to the baseline.

**Figure 2:**
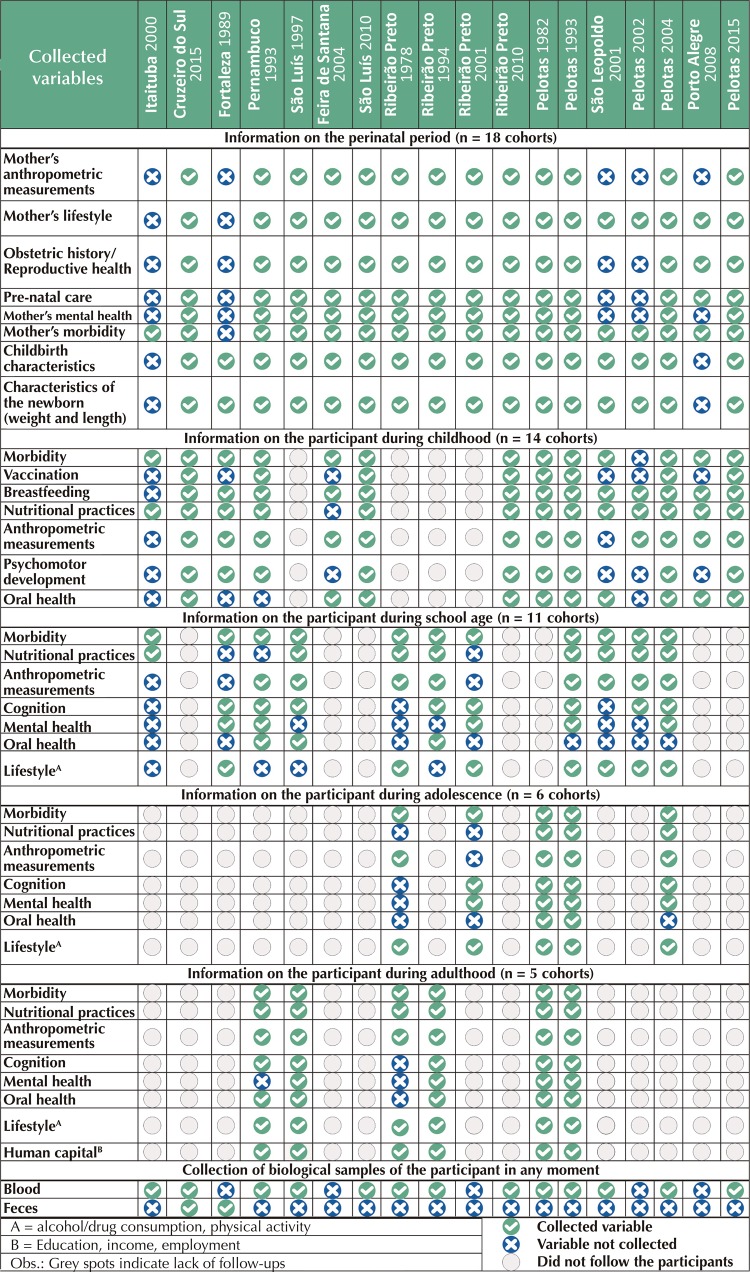
Variables collected by the selected cohorts in each phase of the life cycle.

Regarding the explored variables, all cohorts presented contextual measures (socioeconomic, environmental and domestic variables) in at least one of the follow-ups. The most frequent information collected in the perinatal period were characteristics of the moment of delivery, of the newborn, morbidity and life habits of the pregnant woman, while the least frequent was the mental health of the mother. The most investigated characteristics of the participant in childhood were morbidity, dietary practices and breastfeeding, and the least frequent was vaccination. The cohorts that followed up with the participants at school age collected more frequently morbidity data and less frequently, information on oral health. Five cohorts followed participants in adolescence^21,23,25,26,29^ and investigated more frequently morbidity and lifestyle data. Six cohorts followed participants in the early adulthood^17,18,21,22,25,26^, with data collection of morbidity, dietary practices, anthropometric measurements, cognition, lifestyle and human capital. The information collected less frequently in adulthood was mental health. Of the total cohorts, 14 collected biological material (blood and/or feces)^14^ at some point in the life cycle (Figure 2).

**Figure 3:**
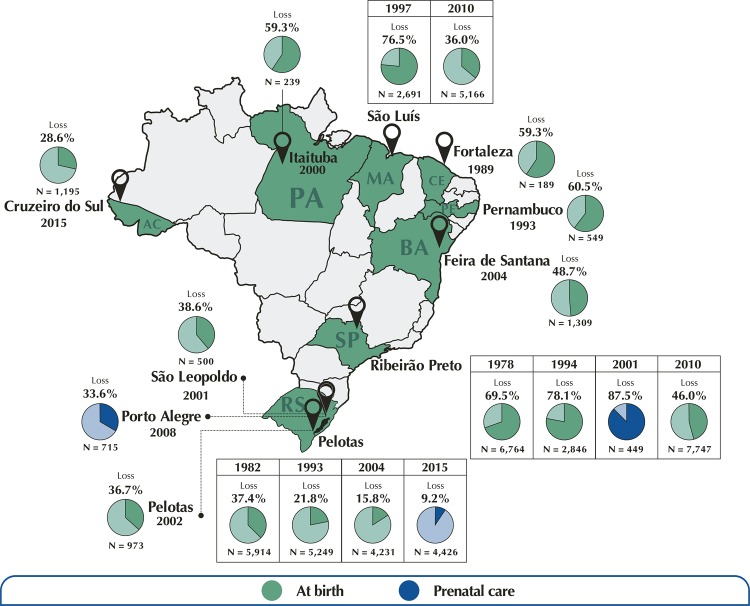
Map of Brazil with geographical distribution of the selected cohorts.

Figure 3 shows the geographical distribution of cohorts in Brazil. The Northern region included two cohorts. The first began in 2000 in the municipality of Itaituba, in the state of Pará^14^; it included 239 children born between 2000–2001 and carried through three additional follow-ups (at ages four, six, and between eight and ten) for mercury exposure analysis. Loss at the last follow-up regarding this cohort was 59.3% in relation to the number of baseline participants. The most recent cohort of the Northern region was conducted in the state of Acre, known as the Maternal-Infant study in Acre (Mina), which began between July 2015 and June 2016 in the municipality of Cruzeiro do Sul^15^. The Mina cohort consisted of all live birth infants living in the urban area of the municipality (n = 1,195), of which 588 had data collected in the prenatal period (up to the second trimester of pregnancy). This cohort performed three follow-ups with the children so far (at 6, 12 and 24 months) and the loss of follow-up at 24 months was 28.6%.

In the Northeastern region, five cohorts were identified. The cohort of Fortaleza, in Ceará, began in 1989 and included 189 children born in the favela of Gonçalves Dias^16^. These children were monitored every three months during their first two years, in the period between 1989 and 1993, for records of diarrheal diseases, dietary information and anthropometric measurements. This cohort performed a total of nine follow-ups (at 3, 6, 9, 12, 15, 18, 21 and 24 months and between 6 and 12 years of age), with loss at the last follow-up of 59.3%. The cohort conducted in five municipalities of Pernambuco (Água Preta, Catende, Joaquim Nabuco, Palmares and Ribeirão), started in 1993, included 549 live birth infants between January 1993 and August 1994^17^. This cohort performed eight follow-ups (at 2, 4, 6, 9, 12 and 24 months, at 8 and 18 years) and had a loss in the last follow-up of 60.5% of the participants. Two population-based cohorts were identified in the city of São Luís, Maranhão. The first, started in 1997, included 2,691 single live birth infants and carried through two follow-ups with participants (between 7 and 9 years and between 18 and 19 years), with loss in follow-up between 18 and 19 years of age of 75.3%^18^. The second cohort was initiated in 2010, known as Brisa São Luís, and included 5,166 live birth infants at baseline, of which 1,447 had data collected in prenatal care^20^. To date, Brisa São Luís has monitored children between 13 and 30 months, with 36% of losses compared to baseline. In Bahia, there is a cohort in the municipality of Feira de Santana since April 2004^19^; it included 1,309 live birth infants, performed ten follow-ups with the participants (at 1, 2, 3, 4, 5, 6, 9 and 12 months, at 3 years and 6 years) and had a loss of 48.7% in the last follow-up.

In the Southeast region, four cohorts were identified, all in the city of Ribeirão Preto, in the state of São Paulo. Three were population-based, started in 1978^21^, 1994^22^ and 2010^24^, and one was hospital-based, started in 2001^23^. The Ribeirão Preto cohort started in 1978 included 6,764 single live birth infants, performed three follow-ups (between 8 and 11 years old, at age 18 – only for male participants – and between ages 23 and 25) and had 69.5% of loss in the last follow-up. The one started in 1994 included 2,846 single live birth infants and carried through two follow-ups with participants (between 9 and 11 years and between 22 and 24 years), with loss in follow-up between 78,1 and 19 years of age of 75.3%. The cohort started in 2010, known as Brisa Ribeirão Preto, included 2,443 single live birth infants of that year, of which 1,417 had data collected in the prenatal period. To date, it has monitored children between 13 and 30 months of life, with loss of follow-up of 46%. The Ribeirão Preto cohort started in 2001, known as the Gesta-Álcool Project, monitored 449 pregnant women in the third trimester of pregnancy and conducted two follow-ups with the participants (between 6 and 7 years and between 11 and 12 years), with a loss of 87.5% in the last follow-up.

In the Southern region of the country, seven cohorts were identified, all in the state of Rio Grande do Sul. We highlight the population-based cohorts of Pelotas, given the high number of individuals recruited, the greater number of follow-ups with the participants and for having followed their participants from birth to adolescence and early adult life. Of the 345 articles selected in this systematic review, 75% corresponded to publications derived from these cohorts. The first cohort of Pelotas was initiated in 1982 and involved 5,914 children, with nine follow-ups to date (at ages 1, 2, 4, 13, 15, 18, 19, 23 and 30)^25^. Subsequent and still ongoing cohorts involved 5,249 births in 1993 with 11 follow-ups (at 1, 3 and 6 months, 1, 4, 6, 9, 11, 12 to 13, 15 and 18 years)^26^; 4,231 births in 2004 with seven follow-ups (at 3 months, 1, 2, 4, 5, 7 and 11 years)^29^ and, in the most recent cohort, 4,226 prenatal recruitments in 2015 with four follow-ups to date (at birth, 3 months, 1 and 2 years). The losses in the last follow-up of the Pelotas cohorts of 1982, 1993, 2004 and 2015 were, respectively, 37.4%, 21.8%, 15.8% and 9.2%. The other three cohorts identified were the one in São Leopoldo, started in 2001^27^, in Pelotas started in 2002^28^ and in Porto Alegre started in 2008^30^. The São Leopoldo cohort recruited 500 mother-child pairs between October 2001 and June 2002 and carried out four follow-ups with the participants (at 6 months, 12 months, 4 years and 7 to 8 years), with loss at the last follow-up of 38.6%. The Pelotas cohort included all 2,741 live birth infants between September 2002 and May 2003 in the urban area of the municipality and followed a random sample of 30% (n = 973) at 1, 3 and 6 months of life and at 8 years of age, with a loss of 36.7% at the last follow-up. The Porto Alegre cohort started with 715 pregnant women recruited in the third week of pregnancy and performed three follow-ups (from 6 to 9 months, 12 to 16 months and 2 to 3 years), with 33.6% loss at the last follow-up.

## DISCUSSION

The Brazilian cohorts identified, initiated in the prenatal or birth period, were prospective and collected primary data. To date, few have followed participants in adolescence (n = 6) and adulthood (n = 5). Six cohorts stood out by the largest number of baseline participants, the comprehensive approach to the health of children, adolescents and adults and by accompanying participants in more phases of the life cycle: the Pelotas birth cohorts of 1982^25^, 1993^26^ and 2004^29^, the Ribeirão Preto cohorts of 1978^21^ and 1994^22^ and the São Luís cohort of 1997^18^. The losses of follow-up were related to not knowing the whereabouts of the participants, to internal migration and refusals to further participation. The most explored variables were related to sociodemographic, environmental and domestic family data, maternal morbidity and lifestyle, additional morbidity data, breastfeeding and nutritional practices during infancy, morbidity during school age, morbidity and lifestyle during adolescence and aspects of integral health during adulthood.

In high-income countries, prenatal and birth cohorts, in addition to accompanying their participants for a prolonged time, are not limited to single-generation individuals; it also includes their descendants^32,33^, allowing for intergenerational analyses. On the other hand, the prenatal and birth cohorts of middle and low-income countries frequently present difficulties related to lack of financial resources, early death of children and internal migration, and infrequent follow-up of participants in more advanced stages of the life cycle^12^.

Some methodological characteristics and difficulties of the identified cohorts were observed. Most monitored two phases of the life cycle (perinatal period and childhood). Although there is a requirement for immediate results to satisfy and stimulate the funders and participants of the study, it is known that the most important results from the perspective of the life cycle are those that analyze the influence of early exposures on health and well-being in later stages of life and with repeated measures at different stages of an individual’s life^34^. Sample size and follow-up losses were weak points of most identified cohorts. Reducing the loss of follow-up over time represents a challenge, since differential losses between exposed and unexposed groups tend to introduce biases in cohort studies^5,6^.

The development process of cohort studies is challenging. The implementation of a cohort requires close collaboration between the institutions involved, health professionals and families, as well as laborious logistics for the correct execution of the phases of the study (preparation of data collection instruments, recruitment and training of study personnel, pilot phase, monitoring of participants), including quality control procedures in all stages^35,36^. In addition to this investment of time, effort and intellectual contribution, keeping a cohort study running is quite costly. The adequacy of funding might determine the timespan of the study. Cohort studies are often funded by government agencies and international organizations, and therefore are vulnerable to political and governmental priorities, or by local research foundations that do not guarantee the sustainability and availability of long-term financial resources^37^.

The development of cohorts in Brazil is important for several reasons. New exposures resulting from demographic, epidemiological, nutritional and institutional transitions in the country create a demand for new investigations on its effects on the health of the Brazilian population.^38^ The possibility of verifying the nature of the associations between exposures and outcomes in different contexts and comparing results between existing cohorts is another advantage^13,39,40^. Brazilian cohorts have the potential to further contribute the scientific capacity of the country, with the training of researchers and improvement of the process of collecting and analyzing data^11^, in addition to assisting decision makers in the analysis of health determinants and in strengthening the health care provided^41^.

In view of the importance of cohorts initiated in the prenatal period and at birth in Brazil, the dichotomy between the development of new cohorts or investment in existing cohorts is discussed. International authors advocate the establishment of new population-based cohorts in countries with socioeconomic and racial/ethnic diversity, with annual follow-ups and evaluation of exposures of greater local relevance for multiple health outcomes^7^. This argument is justified by providing opportunities for collection of new risk information and recruiting underrepresented participants in existing studies, but faces serious budgetary challenges required by cohorts of this size^42^. On the other hand, other authors^43,44^ argue that representativeness is not necessary to determine the relationship between environmental or genetic factors and the risk of disease. These scholars suggest strategies for combining data from existing cohorts^44^ and with the formation of national and international cohort consortia^43^. A systematic review study of European cohorts concluded that a network of coordinated cohort studies, following a harmonized general protocol with a perspective of joint interpretation of data, would increase the scientific impact and international collaboration interest in these researches^45^.

The cohorts with a representative population of Pelotas, Ribeirão Preto and São Luís correspond to the birth cohorts with the greatest potential to study biological, behavioral, social and genetic precursors of chronic diseases in Brazil. The ones in Pelotas are the largest population-based birth cohort studies in low- and middle-income countries with nearly four decades of work, more than 20,000 individuals studied throughout life, regular assessments in childhood and adolescence, and smaller rates of loss at follow-up. The birth cohorts of Ribeirão Preto and São Luís, initiated in partnership with the same group of researchers, studied about 17,000 and 7,000 individuals, respectively, and foresee more follow-ups with their participants. Located in a region with distinct sociocultural characteristics and lacking research, the Mina cohort, in Cruzeiro do Sul, Acre, also has the potential to contribute to life cycle studies in Brazil, since it is the first cohort initiated in with an integral approach to children’s health in the Amazon region.

In conclusion, this review identified cohorts initiated in the prenatal period and at birth in Brazil with potential for life course epidemiology studies. These cohorts differ in sample size, number of follow-ups and phase of the life cycle in which individuals were monitored, as well as rates of loss of follow-up. It is recommended that the Brazilian cohorts initiated in the prenatal period and at birth have enough participants to perform analyses of the effect of early determinants on health outcomes in the short, medium and long term. In addition, in order to expand the epidemiology analyses of the life course in Brazil, it is necessary that the existing cohorts contemplate different social contexts and follow-ups with the participants at different stages of life.
