# Supplementary material for: Brazilian cohorts with potential for life-course studies: a scoping review
Source: Rev Saude Publica. 2020 May 15;54:48. doi: 10.11606/s1518-8787.2020054001825 (PMC7234216; doi:10.11606/s1518-8787.2020054001825)
Supplement: Translation

# Translation: Coortes no Brasil com potencial para estudos do ciclo vital: uma revisão de escopo

**Authors:** Waleska Regina Machado Araujo, Iná S. Santos, Naercio Aquino Menezes, Maria Thereza Costa Coelho de Souza, Antonio Jose Ledo Alves da Cunha, Alicia Matijasevich

**Affiliations:** I Brasil Universidade de São Paulo. Faculdade de Medicina. Programa de Pós-Graduação em Saúde Coletiva. São Paulo, SP, Brasil; II Brasil Pontifícia Universidade Católica do Rio Grande do Sul. Programa de Pós-Graduação em Pediatria e Saúde da Criança. Porto Alegre, RS, Brasil; III Brasil Universidade de São Paulo. Faculdade de Economia e Administração. Departamento de Economia. São Paulo, SP, Brasil; IV Brasil Universidade de São Paulo. Instituto de Psicologia. Departamento de Psicologia da aprendizagem, do desenvolvimento e da personalidade. São Paulo, SP, Brasil; V Brasil Universidade Federal do Rio de Janeiro. Faculdade de Medicina. Departamento de Pediatria. Rio de Janeiro, RJ, Brasil; VI Brasil Universidade de São Paulo. Faculdade de Medicina. Departamento de Medicina Preventiva. São Paulo, SP, Brasil

**Keywords:** Estudos de Coortes, Desenvolvimento Infantil, Desenvolvimento da Saúde, Epidemiologia Analítica, Bibliometria, Revisão Sistemática

## Abstract

**OBJETIVO:**

Identificar as coortes brasileiras iniciadas no período pré-natal ou no nascimento, descrever suas características e as variáveis exploradas, além de mapear as coortes com potencial para se estudar os determinantes precoces de saúde e doença e o risco de adoecer em etapas posteriores do ciclo vital.

**MÉTODOS:**

Realizou-se uma revisão de escopo. A busca dos artigos foi realizada nas bases de dados PubMed e Biblioteca Virtual em Saúde em 16 de junho de 2018. Os descritores utilizados foram [(((“Child” OR “Child, Preschool” OR “Infant” OR “Infant, Newborn”) AND (“Cohort Studies” OR “Longitudinal Studies”)) AND “Brazil”)]. Os critérios de inclusão foram coortes brasileiras que iniciaram a linha de base no período pré-natal ou no nascimento e com pelo menos dois acompanhamentos com os participantes. Foram excluídas as coortes cujos acompanhamentos foram restritos ao primeiro ano de vida, as que não abordaram aspectos biológicos, comportamentais e psicossociais e também aquelas com coleta de informações em um único estágio do ciclo vital.

**RESULTADOS:**

A etapa de busca identificou 5.010 artigos. Foram selecionadas 18 coortes para a síntese descritiva. A mediana do número de participantes na linha de base foi 2.000 indivíduos e a mediana de idade no último acompanhamento foi 9 anos. A perda amostral no último acompanhamento variou de 9,2 a 87,5%. A maioria das coortes realizou acompanhamentos em duas fases do ciclo vital (período perinatal e infância). A região Sul contemplou o maior número de coortes. As principais variáveis coletadas foram sociodemográficas e ambientais da família, aspectos de morbidade, práticas alimentares e estilo de vida dos participantes.

**CONCLUSÕES:**

Recomenda-se a continuidade dessas coortes, a abordagem de diferentes contextos sociais e a realização de acompanhamentos com os participantes em diferentes fases do ciclo vital para o fortalecimento e ampliação das análises de epidemiologia do ciclo vital no Brasil.

## INTRODUÇÃO

A epidemiologia do ciclo vital (ECV) estuda os processos biológicos, comportamentais e psicossociais que ocorrem ao longo da vida dos indivíduos e que ligam a saúde do adulto e o risco de adoecer a exposições físicas ou psicossociais que aconteceram durante a gravidez, infância, adolescência, nas etapas precoces da vida adulta ou através de gerações^1^. O seu propósito é construir e testar modelos teóricos que postulem caminhos que liguem as exposições em diferentes fases do curso da vida a resultados posteriores de saúde^2^. Esses modelos exploram o tempo e as interações de exposições biológicas e psicossociais para identificar riscos e processos de proteção ao longo do ciclo vital^3,4^.

Os estudos de coorte, por acompanharem seus participantes desde a exposição até a ocorrência do desfecho de interesse, são uma ferramenta valiosa para a ECV^5^. Esses estudos são conduzidos geralmente para a avaliação da história natural de doenças, estimação de medidas de frequência de doenças (incidência), identificação dos fatores de risco de agravos de saúde, prognóstico e análise de sobrevida^6,7^. As coortes que coletam informações em diferentes estágios da vida de seus participantes e com desfechos em médio e longo prazo são ótimas para testar modelos explicativos da ECV^8,9^, sendo possível explorar modelos de acumulação de risco e períodos críticos, além de investigar os fatores mediadores e modificadores da associação entre exposições e desfechos em estudo^2,3^.

As coortes que se iniciam no período pré-natal ou no nascimento oferecem uma oportunidade única para avaliar a influência de exposições precoces sobre a saúde ao longo da vida^4^. Elas estão principalmente concentradas nos países de alta renda e se diferenciam das coortes realizadas em países de renda média e baixa pelo maior número de acompanhamentos, seguimento dos participantes por períodos mais prolongados e maior sofisticação das medidas realizadas^10^. Essas coortes, em países de média e baixa renda, oferecem contribuições valiosas em virtude da diversidade de exposições e das diferentes estruturas de determinação de doenças^11^. Em países como o Brasil, com diversidade cultural e desigualdade regional, podem oferecer contribuições tão valiosas quanto as coortes de países de alta. No entanto, não estão mapeadas as lacunas de pesquisa desses estudos no país, sendo oportuna uma síntese de informações com as características e desfechos estudados pelas principais coortes desse tipo para se avançar nos estudos de epidemiologia do ciclo vital no território nacional.

O presente estudo tem como objetivo descrever os estudos de coorte iniciados no período pré-natal ou no nascimento realizados no Brasil, com potencial para estudar os determinantes precoces de saúde e doença e o risco de adoecer em etapas posteriores do ciclo vital. Por meio de uma revisão de escopo, foi possível descrever as principais características metodológicas dessas coortes, as variáveis estudadas, a localização no território nacional e as necessidades relacionadas à pesquisa epidemiológica do ciclo vital no Brasil.

## MÉTODO

Realizou-se uma revisão de escopo para identificar e mapear as evidências disponíveis sobre coortes brasileiras com potencial para estudos de epidemiologia do ciclo vital. Essa revisão é conduzida para resumir e divulgar os resultados da pesquisa e reconhecer lacunas de pesquisa na literatura existente^12^. Seguimos as seguintes etapas: identificação da questão de pesquisa (“Quais as características das coortes brasileiras iniciadas no nascimento e no período pré-natal com potencial para estudar os determinantes precoces de saúde e doença e o risco de adoecer em etapas posteriores do ciclo vital?”), identificação dos artigos relevantes, seleção dos estudos, mapeamento dos dados e compilação, resumo e relato dos resultados. A busca dos artigos foi realizada nas bases de dados eletrônicas PubMed e Biblioteca Virtual em Saúde (BVS). Os descritores foram selecionados no *Medical Subject Headings* (MeSH) com a seguinte combinação: [(((“Child” OR “Child, Preschool” OR “Infant” OR “Infant, Newborn”) AND (“Cohort Studies” OR “Longitudinal Studies”)) AND “Brazil”)]. A estratégia de busca foi adaptada para cada base de dados. Foram considerados na revisão todos os artigos publicados até a data de 16 de junho de 2018.

Foram incluídos na revisão os estudos com delineamento de coorte realizados no Brasil que iniciaram a linha de base do estudo no período pré-natal ou no nascimento e com pelo menos dois acompanhamentos com os participantes. Não foram incluídos os que descrevem só a linha de base e não os acompanhamentos realizados. Para atender ao conceito de ECV, foram excluídas aquelas coortes cujos acompanhamentos foram restritos ao primeiro ano de vida, as coortes que não abordaram aspectos biológicos, comportamentais e psicossociais e as coortes com coleta de informações em um único estágio do ciclo vital. Foram considerados estágios do ciclo vital o período perinatal (entre a 22ª semana de gestação e os seis dias de vida), infância (entre a primeira semana de vida e os seis anos de idade), idade escolar (entre 6 e 12 anos), adolescência (entre 12 e 18 anos) e vida adulta (18 anos ou mais)^13^. Dois revisores realizaram a seleção dos títulos e resumos dos artigos e fizeram a leitura do texto completo dos artigos elegíveis. Como a revisão de escopo objetivou mapear as evidências sobre um tópico relevante, uma avaliação da qualidade das evidências incluídas na revisão não foi necessária^12^. Por fim, a partir da interpretação dos resultados da presente revisão, foram apresentadas recomendações para se avançar nos estudos de epidemiologia do ciclo vital no Brasil.

As seguintes informações foram extraídas dos estudos selecionados: localização geográfica da coorte (cidade, estado da federação, região); ano de início da coorte (linha de base); tipo de coorte conforme a idade dos participantes na linha de base, dividido em nascimento (recrutamento dos participantes iniciado no momento do nascimento) e pré-natal (recrutamento iniciado durante a gestação); tipo de coorte quanto ao momento de ocorrência da exposição e do desfecho e o início do estudo, divido em prospectiva (os dados de exposição foram coletados no início do estudo e o desfecho ainda não ocorreu), retrospectiva (a exposição e o desfecho foram definidos com base nos dados que ocorreram antes do início do estudo, ambos no passado) e ambispectiva (os dados de exposição foram coletados no passado e o desfecho ainda não ocorreu); tipo de dados utilizados na coorte, divididos em primários (dados coletados pela primeira vez, para atender às necessidades da coorte em andamento) e secundários (informações obtidas de dados previamente coletados, cuja coleta não foi por ocasião da pesquisa); base de referência da mostra, dividida em hospitalar (indivíduos de um hospital ou centro de saúde) e populacional (representativa ou de conveniência); número de participantes na linha de base; número de participantes no último acompanhamento; idade média dos participantes no último acompanhamento; número total de acompanhamentos realizados com os participantes; idade média dos participantes em cada acompanhamento; principais informações coletadas em cada fase do ciclo vital.

Esses dados foram complementados com informações extraídas de publicações da lista de referências dos artigos selecionados, de páginas eletrônicas dos estudos de coortes, por contato via endereço eletrônico com pesquisadores dos estudos e consultas a bancos de dissertações e teses. Por fim, realizou-se uma análise descritiva das principais características das coortes incluídas na revisão.

## RESULTADOS

### Resultados da Busca e Seleção dos Artigos

A etapa de busca identificou 5.010 artigos nas bases de dados, sendo excluídos 1.678 artigos duplicados. Após leitura dos títulos e resumos, foram excluídos 2.904 artigos por não atenderem aos critérios de elegibilidade. Após leitura do texto completo, foram identificados 428 artigos de 79 estudos de coorte realizadas no Brasil iniciadas no período pré-natal ou no nascimento e com pelo menos dois acompanhamentos com os participantes. Dessas 79 coortes, foram excluídas 41 com acompanhamentos restritos ao primeiro ano de vida e cinco que não abordaram aspectos biológicos, comportamentais e psicossociais. Devido ao interesse em analisar coortes que exploram os efeitos de exposições que acontecem no período perinatal, infância, adolescência e em fases posteriores do ciclo de vida, foram excluídas quinze coortes com coleta de informações em um único estágio do ciclo vital. Ao final, foram selecionadas 18 coortes (n = 345 artigos). O fluxograma das etapas da revisão está ilustrado na Figura 1.

**Figura 1:** Fluxograma da etapa de busca de seleção de artigos da revisão sistemática.

### Descrição das Coortes Selecionadas

Das 18 coortes identificadas^14^, 15 iniciaram-se no nascimento^14^ e três coortes iniciaram-se no período pré-natal^23,30,31^. Todas as coortes foram prospectivas e coletaram dados primários. Dez coortes^15,18,20^ tiveram uma amostra representativa da população de referência na linha de base, sete coortes^14,16,17,19,27,28,30^ utilizaram uma amostra de conveniência e uma única coorte foi de base hospitalar^23^. O número de participantes na linha de base variou de 189 a 7.747 indivíduos (mediana de 2.000). A média de idade no último acompanhamento variou de 2 a 30 anos (mediana de 9 anos) e o número de acompanhamentos variou de 2 a 11 (mediana de 4), conforme observado na Tabela 1. A perda amostral no último acompanhamento das coortes variou de 9,2 a 87,5% (mediana de 42,0%), sendo as principais dificuldades relatadas pelos autores a não localização dos participantes, perda de contato telefônico e mudança de endereço.

**Tabela 1:** Características das 18 coortes selecionadas, por região geográfica do Brasil.

Localização da coorte	Ano da coorte na linha de base	Idade dos participantes na linha de base	Base de referência da amostra	Número de participantes na linha de base	Número de participantes no último acompanhamento	Idade média no último acompanhamento (anos)	Número total de acompanhamentos	Perda de acompamanhemento^1^
Região Norte								
Itaituba – PA^14^	2000–2001	Nascimento	Amostra da população	239	90	8	3	59,3
Cruzeiro do Sul – AC^15^	2015–2016	Nascimento	População representativa urbana	1.195	853	2	3	28,6
Região Nordeste								
Fortaleza – CE^16^	1989–1993	Nascimento	Amostra da população	189	77	9,6	9	59,3
Municípios de Pernambuco^17^	1993–1994	Nascimento	Amostra da população	549	217	18	8	60,5
São Luís – MA^18^	1997–1998	Nascimento	População representativa	2.691	664	18	2	75,3
Feira de Santana – BA^19^	2004–2005	Nascimento	Amostra da população	1.309	672	6	10	48,7
São Luís – MA^20^	2010	Nascimento	População representativa	5.166	3.306	2	2	36
Região Sudeste								
Ribeirão Preto – SP^21^	1978–1979	Nascimento	População representativa	6.764	2.063	24	3	69,5
Ribeirão Preto – SP^22^	1994	Nascimento	População representativa	2.846	622	23	2	78,1
Ribeirão Preto – SP^23^	2001	Pré-natal	Hospitalar	449	56	12	2	87,5
Ribeirão Preto – SP^24^	2010	Nascimento	População representativa	7.747	4.182	2	2	46
Região Sul								
Pelotas – RS^25^	1982	Nascimento	População representativa	5.914	3.701	30	10	37,4
Pelotas – RS^26^	1993	Nascimento	População representativa	5.249	4.106	18	11	21,8
São Leopoldo – RS^27^	2001–2002	Nascimento	Amostra da população	500	307	7,5	4	38,6
Pelotas – RS^28^	2002–2003	Nascimento	Amostra da população	973	616	8	4	36,7
Pelotas – RS^29^	2004	Nascimento	População representativa	4.231	3.563	11	7	15,8
Porto Alegre – RS^30^	2008	Pré-natal	Amostra da população	715	475	3,2	3	33,6
2Pelotas – RS^31^	2015	Pré-natal	População representativa	4.426	4.018	2	4	9,2

^1^ Percentual de participantes que não foram seguidos no último acompanhamento em relação à linha de base.

Com relação às variáveis exploradas, todas as coortes apresentaram medidas de contexto (variáveis socioeconômicas, ambientais e domiciliares) em pelo menos um dos acompanhamentos. As informações mais frequentes coletadas no período perinatal foram características do momento do parto, do recém-nascido, morbidade e hábitos de vida da gestante, enquanto a menos frequente foi a saúde mental da mãe da criança. As características mais investigadas do participante na infância foram morbidade, práticas alimentares e aleitamento materno, e a menos frequente foi a vacinação. As coortes que realizaram acompanhamento com os participantes na idade escolar coletaram com maior frequência dados de morbidade e em menor frequência, informações sobre saúde bucal. Cinco coortes acompanharam os participantes na adolescência^21,23,25,26,29^ e investigaram mais dados de morbidade e estilo de vida. Seis coortes acompanharam os participantes na fase precoce da vida adulta^17,18,21,22,25,26^, com coleta de dados de morbidade, práticas alimentares, medidas antropométricas, cognição, estilo de vida e capital humano. A informação menos frequentemente coletada na fase adulta foi saúde mental. Do total de coortes, 14 realizaram coletaram material biológico (sangue e/ou fezes)^14^ em algum momento do ciclo vital (Figura 2).

**Figura 2:** Variáveis coletadas pelas coortes selecionadas em cada fase do ciclo vital.

A Figura 3 apresenta a distribuição geográfica das coortes no Brasil. A região Norte contemplou duas coortes. A primeira teve início no ano de 2000 no município de Itaituba, no estado do Pará^14^, foi formada por 239 crianças nascidas em 2000–2001 e realizou mais três acompanhamentos (aos 4, 6 e entre 8 e 10 anos de idade) para análise de exposição de mercúrio. A perda no último acompanhamento dessa coorte foi de 59,3% em relação ao número de participantes da linha de base. A coorte mais recente da região Norte foi realizada no Acre, conhecida como estudo Materno-Infantil no Acre (Mina), iniciada entre julho de 2015 e junho de 2016 no município de Cruzeiro do Sul^15^. A coorte Mina foi formada por todos os nascidos vivos residentes na zona urbana do município (n = 1.195), dos quais 588 tiveram dados coletados no período pré-natal (até o segundo trimestre de gestação). Essa coorte realizou três acompanhamentos com as crianças até o momento (aos 6, 12 e 24 meses) e a perda de acompanhamento aos 24 meses foi de 28,6%.

**Figura 3:** Mapa do Brasil com distribuição geográfica das coortes selecionadas.

No Nordeste, identificaram-se cinco coortes. A coorte de Fortaleza, no Ceará, teve início no ano de 1989 e incluiu 189 crianças nascidas na favela de Gonçalves Dias^16^. Essas crianças foram acompanhadas a cada três meses durante os dois primeiros anos de vida, no período entre os anos de 1989 e 1993, para registros de doenças diarreicas, informações dietéticas e medidas antropométricas. Essa coorte realizou um total de nove acompanhamentos (aos 3, 6, 9, 12, 15, 18, 21 e 24 meses e entre 6 e 12 anos de idade), com perda no último acompanhamento de 59,3%. A coorte realizada em cinco municípios de Pernambuco (Água Preta, Catende, Joaquim Nabuco, Palmares e Ribeirão), iniciada em 1993, incluiu 549 nascidos vivos entre janeiro de 1993 e agosto de 1994^17^. Essa coorte realizou oito acompanhamentos (aos 2, 4, 6, 9, 12 e 24 meses, aos 8 e aos 18 anos) e teve perda no último acompanhamento de 60,5% dos participantes. Foram identificadas duas coortes de base populacional realizadas no município de São Luís, no Maranhão. A primeira, iniciada no ano de 1997, incluiu 2.691 nascidos vivos únicos e realizou dois acompanhamentos com os participantes (entre os 7 e 9 anos e entre os 18 e 19 anos), com perda no acompanhamento entre os 18 e 19 anos de 75,3%^18^. A segunda coorte foi iniciada em 2010, conhecida como Brisa São Luís, e incluiu 5.166 nascidos vivos na linha de base, dos quais 1.447 tiveram dados coletados no pré-natal^20^. Até o momento, a Brisa São Luís acompanhou as crianças na faixa etária entre 13 e 30 meses, com 36% de perdas em relação à linha de base. Na Bahia, tem-se a coorte no município de Feira de Santana, iniciada em abril de 2004^19^, que incluiu 1.309 nascidos vivos, realizou dez acompanhamentos com os participantes (aos 1, 2, 3, 4, 5, 6, 9 e 12 meses, aos 3 anos e aos 6 anos) e teve perda de 48,7% no último acompanhamento.

Na região Sudeste, foram identificadas quatro coortes, todas no município de Ribeirão Preto, no estado de São Paulo. Três foram de base populacional, iniciadas em 1978^21^, 1994^22^ e 2010^24^, e uma foi de base hospitalar, iniciada em 2001^23^. A coorte de Ribeirão Preto iniciada em 1978 incluiu 6.764 nascidos vivos únicos, realizou três acompanhamentos (entre os 8 e 11 anos, aos 18 anos – apenas para os participantes do sexo masculino – e entre os 23 e 25 anos) e teve 69,5% de perda no último acompanhamento. A de 1994 recrutou 2.846 nascidos vivos únicos, realizou dois acompanhamentos com os participantes (entre os 9 e 11 anos e entre os 22 e 24 anos), com perda de 78,1% no último acompanhamento. A coorte iniciada no ano de 2010, conhecida como Brisa Ribeirão Preto, incluiu 2.443 nascidos vivos únicos do referido ano, dos quais 1.417 tiveram dados coletados no período pré-natal. Até o momento, ela acompanhou as crianças entre 13 e 30 meses de vida, com perda de acompanhamento de 46%. A coorte de Ribeirão Preto iniciada em 2001, conhecida como Projeto Gesta-Álcool, acompanhou 449 gestantes no terceiro trimestre de gestação e realizou dois acompanhamentos com os participantes (entre os 6 e 7 anos e entre os 11 e 12 anos), com perda de 87,5% no último acompanhamento.

Na região Sul do país, identificaram-se sete coortes, todas no estado do Rio Grande do Sul. Destacam-se as coortes de base populacional de Pelotas, pelo elevado número de indivíduos recrutados, pelo maior número de acompanhamentos com os participantes e por terem acompanhado seus participantes desde o nascimento até a adolescência e o início da vida adulta. Dos 345 artigos selecionados na presente revisão sistemática, 75% corresponderam a publicações derivadas dessas coortes. A primeira coorte de nascimentos de Pelotas foi iniciada em 1982 e envolveu 5.914 crianças, com nove acompanhamentos até o momento (aos 1, 2, 4, 13, 15, 18, 19, 23 e 30 anos)^25^. As coortes subsequentes e ainda em curso envolveram 5.249 nascimentos no ano de 1993 com 11 acompanhamentos (aos 1, 3 e 6 meses, 1, 4, 6, 9, 11, 12 a 13, 15 e 18 anos)^26^; 4.231 nascimentos no ano de 2004 com sete acompanhamentos (aos 3 meses, 1, 2, 4, 5, 7 e 11 anos)^29^ e, na coorte mais recente, 4.226 recrutamentos no período pré-natal no ano de 2015 com quatro acompanhamentos até o momento (ao nascimento, 3 meses, 1 e 2 anos)^31^. As perdas no último acompanhamento das coortes de Pelotas de 1982, 1993, 2004 e 2015 foram, respectivamente, 37,4%, 21,8%, 15,8% e 9,2%. As outras três coortes identificadas foram a de São Leopoldo iniciada em 2001^27^, a de Pelotas iniciada em 2002^28^ e a de Porto Alegre iniciada em 2008^30^. A coorte de São Leopoldo recrutou 500 pares mãe-filho entre outubro de 2001 e junho de 2002 e realizou quatro acompanhamentos com os participantes (aos 6 meses, 12 meses, 4 anos e 7 a 8 anos), com perda no último acompanhamento de 38,6%. A coorte de Pelotas incluiu todos os 2.741 nascidos vivos entre setembro de 2002 e maio de 2003 na área urbana do município e acompanhou uma amostra aleatória de 30% (n = 973) aos 1, 3 e 6 meses de vida e aos 8 anos de idade, com perda de 36,7% no último acompanhamento. A coorte de Porto Alegre iniciou-se com 715 gestantes recrutadas na terceira semana de gestação e realizou três acompanhamentos (dos 6 a 9 meses, 12 a 16 meses e 2 a 3 anos), com 33,6% de perda no último acompanhamento.

## DISCUSSÃO

As coortes brasileiras identificadas, iniciadas no período pré-natal ou nascimento, foram do tipo prospectivas e coletaram dados primários. Até o momento, poucas acompanharam os participantes na adolescência (n = 6) e na fase adulta (n = 5). Destacaram-se seis coortes pelo maior número de participantes na linha de base, pela abordagem integral da saúde da criança, do adolescente e do adulto e por acompanhar os participantes em mais fases do ciclo de vida: as coortes de nascimento de Pelotas de 1982^25^, 1993^26^ e 2004^29^, as coortes de Ribeirão Preto de 1978^21^ e 1994^22^ e a coorte de São Luís de 1997^18^. As perdas de acompanhamento relacionaram-se à não localização dos participantes do estudo, ao movimento migratório dentro do próprio país e às recusas. As variáveis mais exploradas foram os dados sociodemográficos, ambientais e domésticos da família, morbidade e estilo de vida maternos, dados de morbidade, aleitamento materno e práticas alimentares na infância, morbidade na idade escolar, morbidade e estilo de vida na adolescência e aspectos de saúde integral na fase adulta.

Nos países de alta renda, é frequente que as coortes de pré-natal e nascimento, além de acompanhar por tempo prolongado seus participantes, não se limitem aos indivíduos de uma única geração, incluindo também seus descendentes^32,33^, permitindo as análises entre gerações. Já as coortes de pré-natal e nascimento de países de renda média e baixa apresentam com frequência dificuldades relacionadas à falta de recursos financeiros, morte precoce de crianças e migração interna, sendo pouco frequente o acompanhamento dos participantes em fases mais avançadas do ciclo vital^12^.

Foram observadas algumas características e dificuldades metodológicas das coortes identificadas. A maioria realizou acompanhamentos em duas fases do ciclo vital (período perinatal e infância). Embora exista a exigência de resultados imediatos para satisfazer e estimular os financiadores e participantes do estudo, é sabido que os resultados mais importantes na perspectiva do ciclo vital são aqueles que analisam a influência das exposições precoces sobre a saúde e bem-estar em estágios posteriores da vida e com medidas repetidas em diferentes fases da vida de um indivíduo^34^. O tamanho da amostra e as perdas de acompanhamento foram pontos fracos da maioria das coortes identificadas. Diminuir a perda de acompanhamento ao longo do tempo representa um desafio, já que perdas diferenciais entre os grupos expostos e não expostos tendem a introduzir vieses nos estudos de coorte^5,6^.

O processo de desenvolvimento dos estudos de coorte mostra-se desafiador. A implantação de uma coorte requer uma estreita colaboração entre as instituições envolvidas, os profissionais de saúde e as famílias, assim como uma logística laboriosa para a correta execução das fases do estudo (elaboração dos instrumentos de coleta de dados, recrutamento e treinamento do pessoal do estudo, fase-piloto, acompanhamento dos participantes), incluindo procedimentos de controle de qualidade em todas as etapas^35,36^. Além desse investimento de tempo, esforço e contribuição intelectual, manter um estudo de coorte em funcionamento é bastante custoso. O financiamento em níveis adequados e em longo prazo pode definir se o estudo poderá se estender no tempo. Frequentemente os estudos de coorte são financiados por agências do governo e organizações internacionais, sendo vulneráveis às prioridades políticas e governamentais, ou por fundações de pesquisa locais que não garantem a sustentabilidade e disponibilidade de recurso financeiro em longo prazo^37^.

O desenvolvimento de coortes no Brasil é importante por diversas razões. As novas exposições decorrentes das transições demográfica, epidemiológica, nutricional e assistencial vivenciadas pelo país demandam novas investigações sobre seus efeitos na saúde da população brasileira^38^. A possibilidade de verificar a natureza das associações entre exposições e desfechos em diferentes contextos e comparar resultados entre coortes existentes é outra vantagem^13,39,40^. As coortes brasileiras têm o potencial de desenvolver a capacidade científica do país, com a formação de pesquisadores e aprimoramento do processo de coleta e análises de dados^11^, além de auxiliar tomadores de decisão na análise de determinantes de saúde e no fortalecimento dos cuidados à saúde prestados^41^.

Tendo em vista a importância de coortes iniciadas no período pré-natal e ao nascimento no Brasil, coloca-se em discussão a dicotomia entre o desenvolvimento de novas coortes ou investimento nas coortes já existentes. Autores internacionais defendem a instauração de novas coortes de base populacional em países com diversidade socioeconômica e racial/étnica, com acompanhamentos anuais e avaliação das exposições de maior relevância local para múltiplos desfechos de saúde^7^. Esse argumento é justificado por oportunizar a coleta de novas informações de risco e recrutar participantes sub-representados em estudos existentes, mas esbarra em sérios desafios orçamentários requeridos por coortes desse porte^42^. Em contrapartida, outros autores^43,44^ argumentam que a representatividade não é necessária para determinar a relação entre fatores ambientais ou genéticos e o risco de doença. Esses últimos sugerem estratégias de combinação de dados de coortes já existentes^44^ e com a formação de consórcios de coorte nacionais e internacionais^43^. Um estudo de revisão sistemática de coortes europeias concluiu que uma rede de estudos de coorte coordenados, seguindo um protocolo geral harmonizado e com perspectiva de uma interpretação conjunta de dados, aumentaria o impacto científico e o interesse de colaboração internacional por essas pesquisas^45^.

As coortes com população representativa de Pelotas, Ribeirão Preto e São Luís correspondem às coortes de nascimento com maior potencial de estudar precursores biológicos, comportamentais, sociais e genéticos das doenças crônicas no Brasil. As de Pelotas são os maiores estudos de coorte de nascimentos de base populacional em países de baixa e média renda, com quase quatro décadas de trabalho, mais de 20.000 indivíduos estudados ao longo da vida, avaliações regulares na infância e adolescência e as taxas mais baixas de perda de acompanhamento dos participantes. As coortes de nascimento de Ribeirão Preto e São Luís, iniciadas em parceria com o mesmo grupo de pesquisadores, estudaram cerca de 17.000 e 7.000 indivíduos, respectivamente, e preveem mais acompanhamentos com seus participantes. Localizada em uma região com características socioculturais distintas e que carece de pesquisas, a coorte Mina, em Cruzeiro do Sul, no Acre, também possui potencial para contribuir para estudos de ciclo de vida no Brasil, uma vez que é a primeira coorte iniciada no nascimento, com abordagem integral da saúde da criança, na região amazônica.

Em conclusão, a presente revisão identificou coortes iniciadas no período pré-natal e ao nascimento no Brasil com potencial para estudos de epidemiologia do ciclo vital. Essas coortes diferem quanto ao tamanho da amostra, número de acompanhamentos e fase do ciclo vital em que os indivíduos foram acompanhados, bem como taxas de perdas de acompanhamentos. Recomenda-se que as coortes brasileiras iniciadas no período pré-natal e no nascimento tenham número de participantes suficiente para realizar análises do efeito de determinantes precoces em resultados de saúde em curto, médio e longo prazo. Além disso, para que se ampliem as análises de epidemiologia do ciclo vital no Brasil, é necessário que as coortes existentes contemplem diferentes contextos sociais e realizem acompanhamentos com os participantes em diferentes fases da vida.
